# Supplementary material for: Ginsenoside Rh2 Ameliorates Atopic Dermatitis in NC/Nga Mice by Suppressing NF-kappaB-Mediated Thymic Stromal Lymphopoietin Expression and T Helper Type 2 Differentiation
Source: Int J Mol Sci. 2019 Dec 4;20(24):6111. doi: 10.3390/ijms20246111 (PMC6940811; doi:10.3390/ijms20246111)
Supplement: Supplementary file 1 [file ijms-20-06111-s001.pdf]

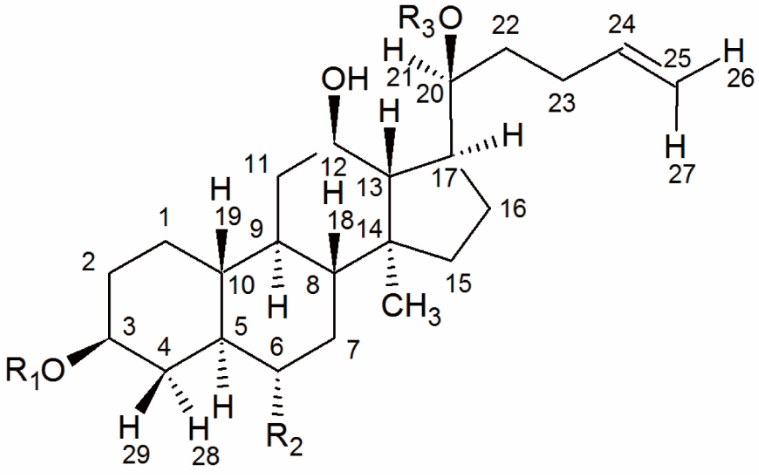

Ginsenoside	R1	R2	R3
Compound K (C-K)	H-	H-	Glc-
F1	H-	HO-	Glc-
F2	Glc-	H-	Glc-
Gypenoside XVII (G17)	Glc-	H-	Glc(1→6)Glc-
Gypenoside LXXV (G75)	H-	H-	Glc(1→6)Glc-
Protopanaxadiol (PPD)	H-	H-	H-
Protopanaxatriol (PPT)	H-	HO-	H-
Rb1	Glc(1→2)Glc-	H-	Glc(1→6)Glc-
Rb3	Glc(1→2)Glc-	H-	Xyl(1→6)Glc-
Rc	Glc(1→2)Glc-	H-	Araf(1→6)Glc-
Rd	Glc(1→2)Glc-	H-	Glc-
Re	H-	Rha(1→2)-Glc-O-	Glc-
Rg1	H-	Glc-O-	Glc-
Rg2	H-	Rha(1→2)-Glc-O-	H-
Rg3	Glc(2→1)Glc-	H-	H-
Rh1	H-	Glc-O-	H-
Rh2	H-	H-	Glc-

Glc : Glucopyranoside; Rha : Rhamnopyranoside; Xyl : Xylopyranoside; Araf : Arabinofuranoside

Figure S1. Chemical structure of ginsenosides.

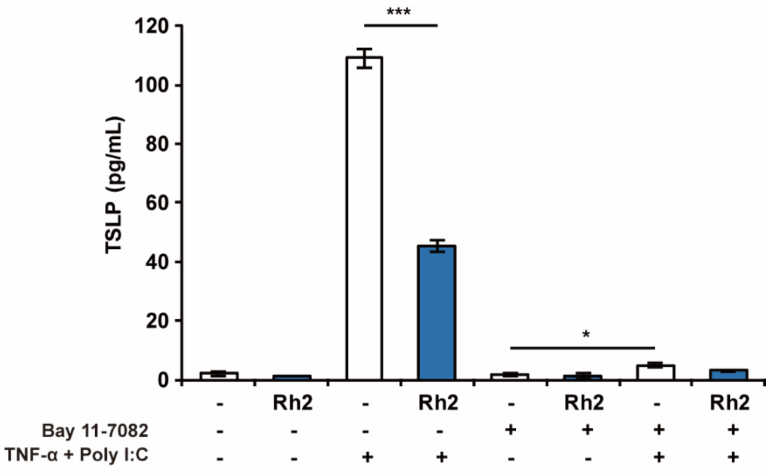

Figure S2. NHKs were treated with 10 μM of Bay 11-7082 for 30 min, followed by treatment with DMSO (vehicle control) or Rh2 (5 μM) while co-stimulating with 20 ng/mL TNF-α and 100 μg/mL Poly I:C for 24 h. Production of TSLP in the cell supernatant was measured by ELISA. *$p < 0.05$, ***$p < 0.001$. $n = 3$.
